# Wheel Slip Control Applied to an Electric Tractor for Improving Tractive Efficiency and Reducing Energy Consumption

**DOI:** 10.3390/s22124527

**Published:** 2022-06-15

**Authors:** Rodnei Regis de Melo, Fernando Lessa Tofoli, Sérgio Daher, Fernando Luiz Marcelo Antunes

**Affiliations:** 1 Federal Institute of Education, Science, and Technology of Ceará, Limoeiro do Norte 62930-000, Brazil; 2 Department of Electrical Engineering, Federal University of São João del-Rei, São João del-Rei 36307-352, Brazil; fernandolessa@ufsj.edu.br; 3 Department of Electrical Engineering, Federal University of Ceará, Fortaleza 60455-760, Brazil; sdaher@dee.ufc.br (S.D.); fantunes@dee.ufc.br (F.L.M.A.)

**Keywords:** agricultural activities, electric tractors, proportional-integral control, slip control

## Abstract

This work presents an automatic slip control solution applied to a two-wheel-drive (2WD) electric tractor. Considering that the slip can be maintained within a specific range that depends on the type of soil, it is possible to increase the tractive efficiency of the electric vehicle (EV). The control system can be easily designed considering only the longitudinal dynamics of the tractor while using simple proportional-integral (PI) controllers to drive the inverters associated with the rear wheels. The introduced solution is tested on an experimental electric tractor prototype traveling on firm soil considering case studies in which the slip control is enabled and disabled. The acquired results demonstrate that the slip control allows for obtaining a more stable performance and reduced energy consumption.

## 1. Introduction

Mechanization is of major importance for increasing productivity in agricultural activities while taking into account sustainability and socio-environmental issues [1]. In this scenario, tractors play an important role in agricultural crop production. Conventional tractors rely on propulsion systems based on internal combustion engines (ICEs). However, electric tractors have been suggested more recently as an interesting alternative for the reduction of fossil fuel consumption and greenhouse gas emissions [2]. Another relevant aspect is the possible association of such electric vehicles (EVs) with distributed generation microsystems that employ clean and renewable energy sources [3]. For instance, an extended-range solar assist plug-in hybrid electric tractor for light agricultural applications is designed in [4].

An extensive analysis of the recent state of the art related to electric and hybrid tractors is presented in [5], which assessed the high-voltage electrification in terms of auxiliary and non-propulsion loads, traction drives, energy storage devices, and implement electrification. The performance evaluation of agricultural electric tractors and their respective control strategies have also been the scope of some works focused on the introduction of accurate numerical models [6]. In this context, efficient energy management strategies combined with proper software and hardware tools are essential to optimize such novel powertrains [7].

A four-wheel-drive (4WD) ICE tractor was converted into a pure EV by the authors in [8] by replacing a two-cylinder diesel engine with a 10 kW three-phase induction motor. The study demonstrated that the overall weight increases, but the relative displacement of the center of gravity (CG) changes only slightly. On the other hand, the energy consumption during traveling and tilling activities could be reduced by up to 70%. An electric tractor motor drive based on brushless dc (BLDC) motors and a double closed-loop proportional-integral-derivative (PID) control system is described in [9]. The life-cycle assessment of electric and ICE tractors is presented in [10], showing that the levelized cost of energy (LCE) is much higher for vehicles based on fossil fuels.

It is also effectively shown in [11] that torque management plays an important role in field traction and road driving conditions. This aspect motivated the development of load torque-based control strategies, as in [12], which employ the well-known particle swarm optimization (PSO) algorithm to determine the best working point aiming at maximum energy conversion efficiency.

Traction performance is an important metric related to agricultural equipment, which defines the draft force of the attached implement and is essential for designing effective tractors [13]. It is worth mentioning that slip control is of major interest for ensuring the operation with maximum tractive efficiency, especially because it tends to decrease when the wheel sleep is outside the range defined between 8% and 15% [14]. According to [15], the longitudinal slip efficiency of driving wheels in off-road vehicles depends not only on the wheel slip, but also on the gross traction force. This work suggests that slip control is a must to maximize slip efficiency. Considering that the slip is a nonlinear function of the ground speed and wheel rotation frequency, while also depending on the internal state variables that are often unknown, an adaptive Kalman filter is used in [16] to estimate the wheel load torque.

The literature presents some solutions for controlling the wheel slip in agricultural tractors. A coupled drive system for electric tractors is assessed in [17], but the authors only performed hardware-in-the-loop (HIL) experiments and no actual field tests. The dual-motor power coupling drive system described in [18] relies on a PSO-based approach for adjusting the system parameters, but this is a somewhat complex solution. The intelligent ballast control system in [19] allows for obtaining active load transfer in electric tractors. The results showed that the mean wheel slip in active ballasting mode was lower than that without ballasting control, but this aspect was only investigated in terms of HIL tests. Sliding mode control (SMC) and incremental proportional-integral (IPI) control were compared in [20] to achieve travel reduction in electric tractors, but the slippage of traction wheels was not analyzed in detail. Similarly, integral sliding mode (ISM) and proportional-integral (PI) control applied in wheel slip control are compared in [21] in terms of simulation results only. In turn, the focus in [22] is given to estimating the optimum slip that leads to the maximum traction and low soil disturbances in unmanned electric tractors, whereas an online slip control algorithm based on SMC is proposed.

A fuzzy-based automatic control system is proposed in [23] for measuring the slip and adjusting the depth of tillage equipment accordingly. The test results of the introduced solution are compared with those obtained from a manual operator control, resulting in a significant decrease in energy consumption. A similar microcontroller-based approach for two-wheel-drive (2WD) tractors is assessed in [24]. The system measures the wheel slip under varying field conditions from the actual and theoretical speeds of the tractor associated with the front and rear wheels. A real-time microcontroller-based embedded system for automatic slip-draft control is proposed in [25], in which the slip is calculated from the differential speed of the front and rear wheels. The performance of an electric all-wheel-drive (AWD) tractor is evaluated in [26], in which the slip does not exceed 15% to measure the traction force under severe conditions.

It is worth mentioning that wheel slip is an important issue that influences the dynamical behavior and performance of tractors in field activities. However, slip control applied in electric tractors still remains relatively unexplored in the literature. In this context, the main contribution of this work is the introduction of an algorithm applied to a slip control system, which allows the electric tractor to operate with high torque and optimized performance in distinct scenarios. Unlike ICE-based tractors, electric powertrains incorporate the possibility of monitoring several variables of interest using sensors, e.g., velocity and rotation speed, among other parameters. Considering that the slip is the main factor responsible for the loss of tractive efficiency, the introduced control approach also incorporates specific characteristics of the application, which include the type of soil. To the best of the authors’ knowledge, this aspect has not been considered in the conception of other similar solutions so far.

In this context, this work proposes a slip control system for increasing the tractive efficiency and minimizing the power losses in an electric tractor, also aiming at increasing the battery bank autonomy. The remainder of this work is organized as follows. Section 2 describes the tractor model, as well as other relevant issues for designing the proposed control system. Section 3 discusses experimental results obtained from a 2WD tractor, in which slip is thoroughly evaluated to demonstrate the effectiveness of the introduced control solution. Section 4 presents the main conclusions.

## 2. Proposed Control System

### 2.1. Tractor Model

**Figure 1:**
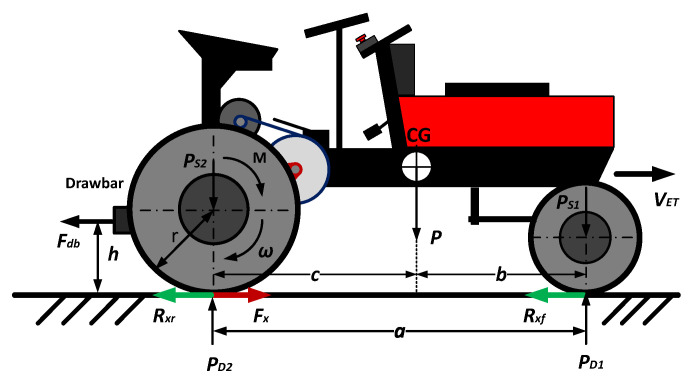
Electric tractor model.

Figure 1 presents the model used in this study, which takes into account only the longitudinal movement of the two rear wheels. Since agricultural tractors often travel at low speeds, the aerodynamic resistance is neglected too. The resultant force associated with the wheel and ground interaction is defined as the drawbar force *F_db_*. Considering that there is a complex wheel–ground interaction, the forces acting on the longitudinal axis can be expressed by Equations (1)–(4).

(1) $$m{\dot{V}}_{ET}=F_{x}-F_{db}-R_{r},$$

(2) $$R_{r}=R_{xf}+R_{xr}=C_{r}P_{D1}+C_{r}P_{D2},$$

(3) $$P_{D1}=\frac{-h\left(F_{db}+m{\dot{V}}_{ET}\right)+cP}{a},$$

(4) $$P_{D2}=\frac{h\left(F_{db}+m{\dot{V}}_{ET}\right)+bP}{a},$$

where *m* is the tractor mass; ${\dot{V}}_{ET}$ is the tractor velocity; *F_x_* is the longitudinal wheel force; *R_r_* is the rolling resistance; *R_xf_* and *R_xr_* are the rolling resistances of the front and rear wheels, respectively; *C_r_* is the tire rolling resistance coefficient; *P_D_*_1_ and *P_D_*_2_ are the front wheel and rear wheel loads, respectively; *a* is the distance between the axles; *b* is the distance from the CG to the front axle; *c* is the distance from the CG to the rear axle; and *h* is the drawbar height.

The longitudinal slip *λ* can be calculated from Equation (5) as a function of the wheel velocity *V_W_*, which in turn can be obtained from Equation (6).

(5) $$\lambda =\frac{V_{W}-V_{ET}}{V_{W}},$$

(6) $$V_{W}=R\omega ,$$

where *R* and *ω* are the wheel radius and angular speed, respectively.

**Figure 2:**
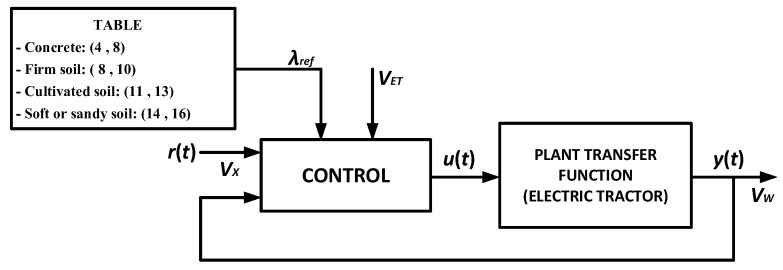
Slip control system applied to an electric tractor.

It is worth mentioning that the wheel velocity and the tractor velocity are useful parameters for calculating the slip. Based on the aforementioned theoretical assumptions and equations that represent the longitudinal model, one can obtain a strategy capable of providing slip control in an electric tractor, while respecting specific characteristics of such an application, which include the type of soil (*λ_ref_*), as shown in Figure 2. Signal *u*(*t*) (control signal) corresponds to the input, whereas *y*(*t*) is the output (controlled signal—*V_W_*) and *r*(*t*) is the reference signal (accelerator pedal—*V_x_*).

**Figure 3:**
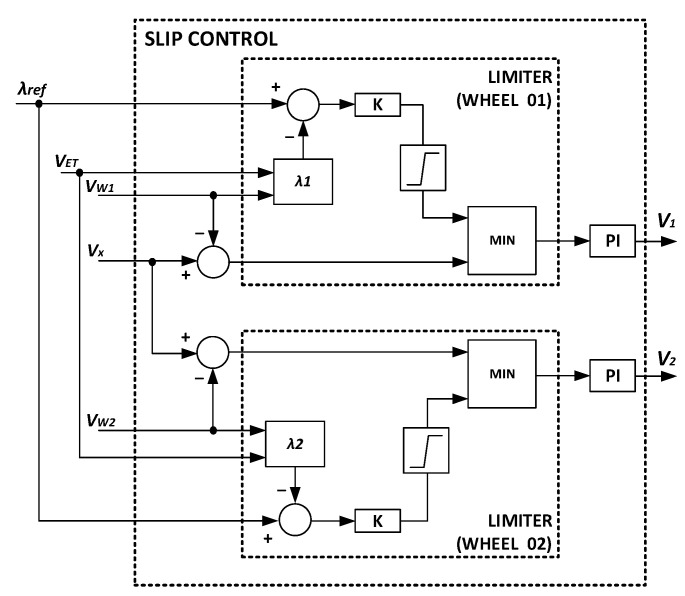
Proposed slip control system.

The proposed control approach relies on the linear representation of the tractor longitudinal dynamics using a PI controller. Figure 3 represents the block diagram of the slip control system, where *V*_1_ and *V*_2_ are the reference velocities applied to the inverter responsible for driving the motors.

The first step consists in obtaining the system model using a parametric identification method based on data measured from the electric tractor. The model parameters are *V_x_* and *V_W_*, which were collected during experimental tests so that it is possible to represent the plant in the form of an auto-regressive with exogenous input (ARX) structure, corresponding to the transfer function given in Equation (7) [27].

(7) $$H\left(z^{-1}\right)=\frac{B\left(z^{-1}\right)}{A\left(z^{-1}\right)}=\frac{b_{1}\left(z^{-1}\right)}{1+a_{1}\left(z^{-1}\right)}.$$

where *A*(*z*^−1^) and *B*(*z*^−1^) are characteristic polynomials represented in terms of their respective coefficients *a*_1_ and *b*_1_.

**Figure 4:**
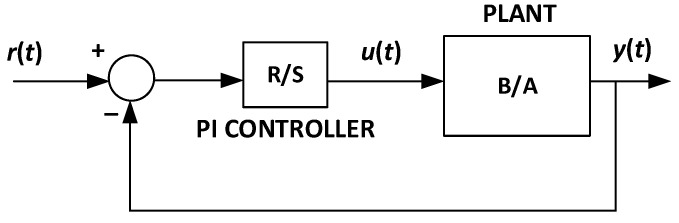
Closed-loop control system based on a PI controller.

Figure 4 shows that the transfer function of a PI controller consists of two polynomials *R*(*z*^−1^) and *S*(*z*^−1^). The polynomials are adjusted so as to meet the system requirements, considering that the controller input is the difference between the reference *r*(*t*) and the measured output *y*(*t*), whereas the controller output is the control signal *u*(*t*). According to [28], the control law of a PI controller is defined by Equation (8). Thus, one can represent *R*(*z*^−1^) and *S*(*z*^−1^) as in Equations (9) and (10), respectively.

(8) $$u\left(t\right)=K\left[\frac{\left(1-z^{-1}\right)+\left(T_{s}/T_{i}\right)}{\left(1-z^{-1}\right)}\right]\left[r\left(t\right)-y\left(t\right)\right].$$

where *K* is the gain, *T_s_* is the sampling period, and *T_i_* is the integral action.

(9) $$R\left(z^{-1}\right)=K\left(1+T_{s}/T_{i}\right)-Kz^{-1}=r_{0}+r_{1}z^{-1},$$

(10) $$S\left(z^{-1}\right)=1-z^{-1}=1-s_{1}z^{-1},\;s_{1}=-1,$$

where *r*_0_, *r*_1_, and *s*_1_ are polynomial coefficients.

The closed-loop performance in terms of the settling time and overshoot is defined by a second-order polynomial corresponding to the discrete representation of a second-order system in the time domain according to Equation (11). Thus, one can define the control law using the pole allocation method. The parameters of polynomials *R*(*z*^−1^) and *S*(*z*^−1^) are calculated from the Diophantine equation corresponding to Equation (12) and its respective coefficients in Equations (13) and (14). It is then possible to obtain the coefficients of the digital PI controller from Equations (15) and (16).

(11) $$H_{CL}\left(z^{-1}\right)=\frac{B\left(z^{-1}\right)R\left(z^{-1}\right)}{A\left(z^{-1}\right)S\left(z^{-1}\right)+B\left(z^{-1}\right)R\left(z^{-1}\right)}=\frac{B\left(z^{-1}\right)R\left(z^{-1}\right)}{P\left(z^{-1}\right)},$$

(12) $$1+\left(a_{1}-1+b_{1}r_{o}\right)z^{-1}+\left(b_{1}r_{1}-a_{1}\right)z^{-2}=1+p_{1}z^{-1}+p_{2}z^{-2},$$

(13) $$p_{1}=a_{1}-1+b_{1}r_{o},$$

(14) $$p_{2}=b_{1}r_{1}-a_{1},$$

(15) $$r_{o}=\frac{p_{1}-a_{1}+1}{b_{1}},$$

(16) $$r_{1}=\frac{p_{2}+a_{1}}{b_{1}}.$$

It is observed that tuning the controller depends on the parameters of the plant (*a*_1_, *b*_1_) and the desired polynomial (closed-loop poles *p*_1_ and *p*_2_). Therefore, one can easily obtain the time-domain representation of the PI controller from Equations (17) and (18).

(17) $$K=-r_{1},$$

(18) $$T_{i}=-\frac{T_{s}r_{1}}{r_{0}+r_{1}}.$$

### 2.2. System Identification

**Figure 5:**
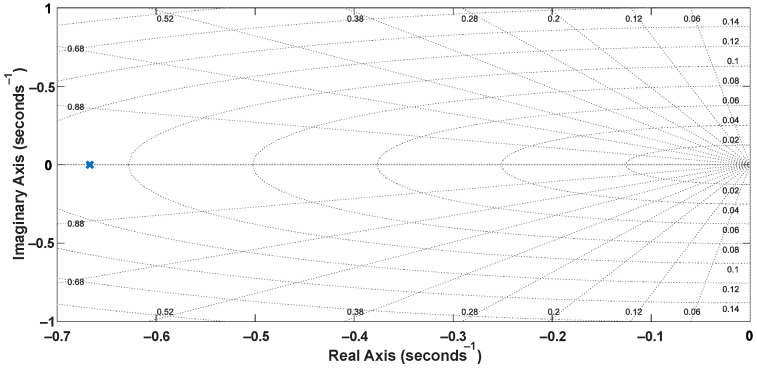
Pole-zero map of the ARX model.

**Figure 6:**
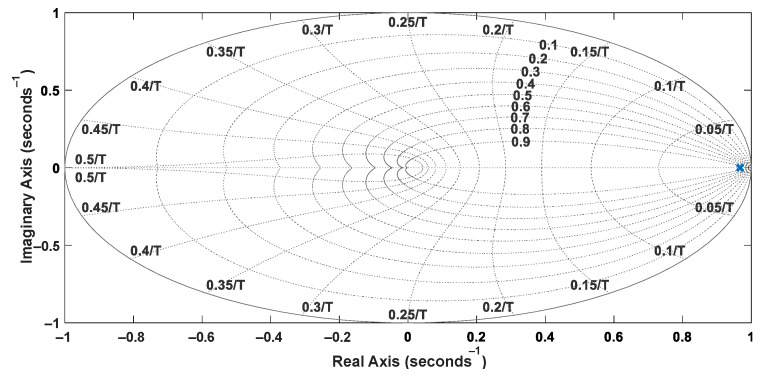
Pole-zero map of the discretized model.

**Figure 7:**
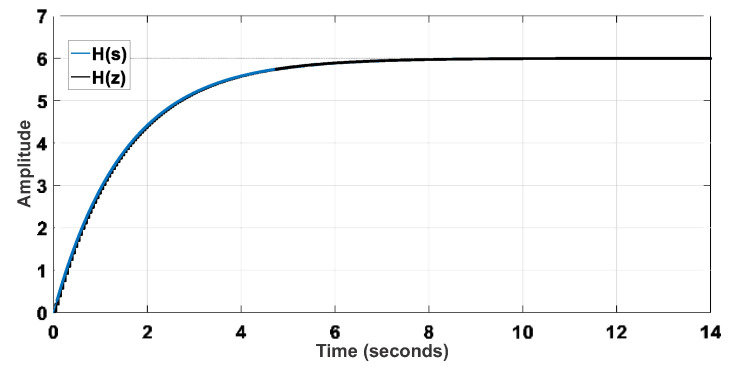
Step response of the plant models.

The system identification was performed experimentally considering a single-input, single-output (SISO) structure involving the reference velocity *V_x_* and the motor shaft velocity *V_W_*. A step signal was applied to the input (*V_x_*) using the acceleration pedal, and the velocities *V_W_*_1_ and *V_W_*_2_ could be measured from analog output signals of the inverters that drive the wheels, also considering a sampling time of 50 ms. Thus, one can obtain Equation (19), whose discretized representation corresponds to Equation (20). The pole-zero maps of the plant model represented in the continuous and discrete domains are shown in Figure 5 and Figure 6, respectively. The step response of the plant models is presented in Figure 7.

(19) $$H\left(s\right)=\frac{4}{s+0.667},$$

(20) $$H\left(z^{-1}\right)=\frac{B\left(z^{-1}\right)}{A\left(z^{-1}\right)}=\frac{b_{1}\left(z^{-1}\right)}{1+a_{1}\left(z^{-1}\right)}=\frac{0.1967}{z-0.9672}.$$

**Figure 8:**
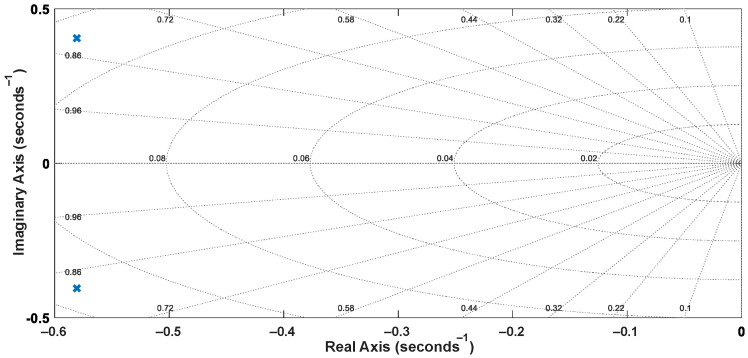
Pole-zero map of the reference model in the continuous domain.

**Figure 9:**
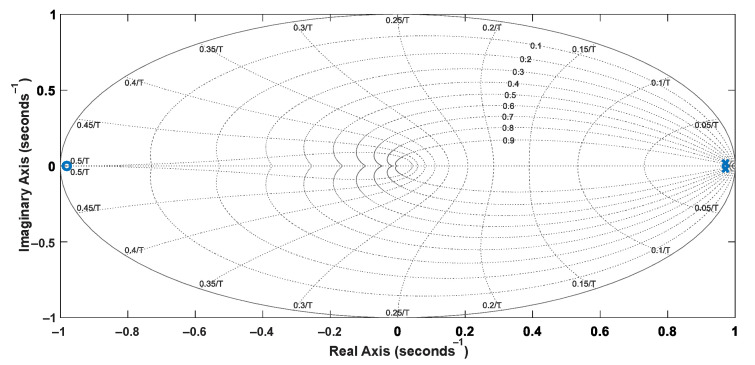
Pole-zero map of the discretized reference model.

**Figure 10:**
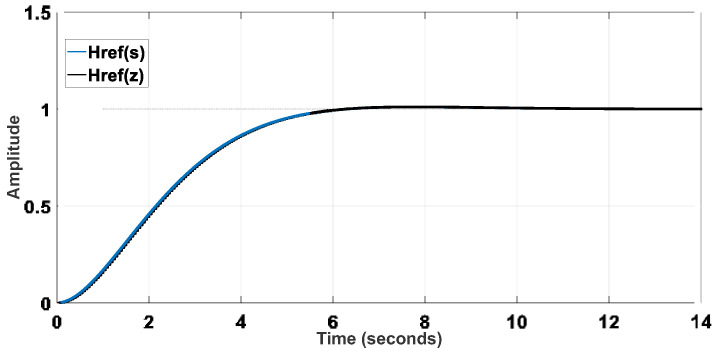
Step response of the reference models.

The reference model can be defined from proper design criteria, resulting in Equation (21), whose discretized form is Equation (22). Then, it is possible to represent the reference model in terms of Figure 8 and Figure 9. The step response of the reference models is also shown in Figure 10.

(21) $$H_{ref}\left(s\right)=\frac{0.5011}{s^{2}+1.161s+0.5011},$$

(22) $$H_{ref}\left(z^{-1}\right)=\frac{0.0006144z+0.0006026}{z^{-1}+1.9423691z+0.9435861}.$$

**Figure 11:**
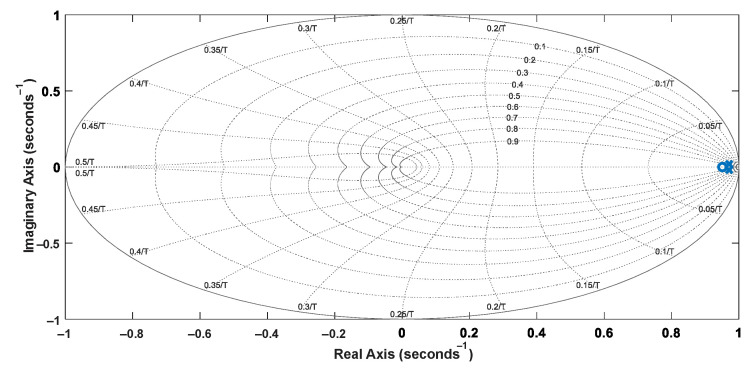
Pole-zero map of the discretized closed-loop model.

**Figure 12:**
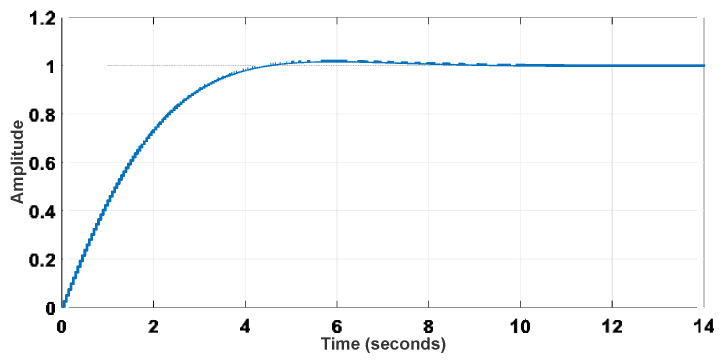
Step response of the closed-loop model.

The discretized transfer function of the closed-loop system is given by Equation (23). It is worth mentioning that the roots of *P*(*z*^−1^) are the closed-loop poles, resulting in *p*_1_ = −1.9423691 and *p*_2_ = −0.9435861. The pole-zero map and the step response of the discretized closed-loop system are shown in Figure 11 and Figure 12, respectively.

(23) $$H_{CL}\left(z^{-1}\right)=\frac{0.02483z-0.02361}{z^{2}-1.9423691z+0.9435861}.$$

From Equations (15) and (16), one can easily obtain the parameters of the digital PI controller, that is, *r*_0_ = 0.1262 and *r*_1_ = −0.12. Similarly, the parameters of the PI controller in the continuous domain can be easily calculated from Equations (17) and (18), resulting in *K* = 0.12 and *T_i_* = 0.9702. Since a limiter is employed in the control system represented in Figure 3, the digital control algorithm was not implemented based on the difference equation. In practice, it was developed from the parameters of the equivalent PI controller in the continuous domain with an anti-windup algorithm based on the guidelines provided in [29].

## 3. Experimental Results

**Figure 13:**
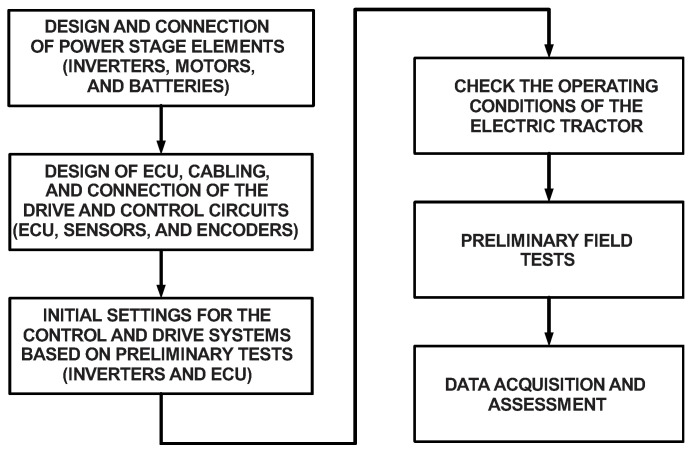
Flowchart representing the design procedure of the electric tractor powertrain and slip control system.

Figure 13 presents a flowchart corresponding to the design procedure of the electric tractor powertrain associated with the proposed slip control. The control system aggregates an electronic control unit (ECU) associated with a digital signal controller and cables connected to the drive and control circuits of the inverters responsible for driving the induction motors. The ECU was embedded in a digital signal programmable interface controller (dsPIC) model dsPIC30F4013, whose 16-bit architecture allows for obtaining high computing performance.

The accelerator pedal comprises a potentiometer as typically used in urban vehicles, which is responsible for obtaining the reference speed of the vehicle. A 360 rotary encoder coupled to the front wheel is also employed to measure the speed. A current sensor connected in series with the battery bank allows for measuring the energy consumption. The system employs a voltage sensor in the ECU to monitor the batteries, as well as encoders and temperature sensors to monitor the induction motors. The battery bank comprises one voltage sensor and one current sensor as well. A more detailed description of the design can be found in [30].

**Figure 14:**
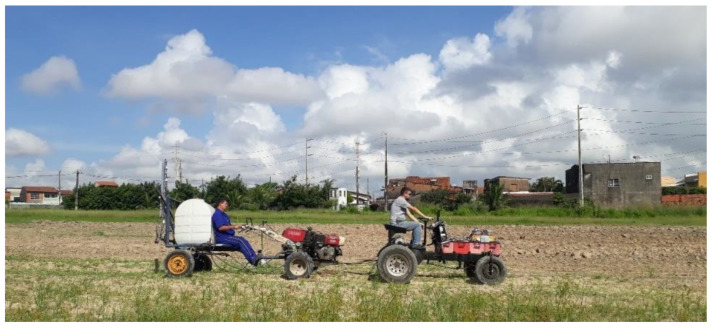
Electric tractor coupled to an agricultural sprayer and a 10.3-kW ICE tractor.

The electric tractor shown in Figure 14 was tested on firm soil with and without the proposed slip control to assess the behavior of several key variables and establish a fair comparison between both approaches. Three trial sessions were performed and repeated three times each considering the following conditions: traction motors at the maximum rotating speed while the slip control is disabled, slip controlled manually by the operator through the pedal, and slip control enabled to adjust the rotating speed automatically.

Each test was performed on a field area with a length of 15 m. The load dragged consisted of an agricultural sprayer supplied with 200 L of water, which had a tank with a capacity of 400 L and was coupled to a 10.3-kW ICE tractor. The maximum speed of the electric motors was set to 800 rpm to ensure an average speed of 0.9 m/s (3.24 km/h) on the traction wheels. The slip reference was set to 10% in the tests with the slip control enabled, this being an adequate value for firm soils. The acquired data were analyzed using several figures of merit, that is, the mean, standard deviation, variance, median, maximum, minimum, symmetry, and kurtosis. If the symmetry and kurtosis coefficients remain between −2 and 2, one can state that the data are normally distributed [31]. The control chart was used to perform a qualitative analysis, considering that such a statistical process control (SPC) tool allows for monitoring and evaluating the results of activities related to mechanized agricultural operations [32].

### 3.1. Test on Firm Soil without Slip Control

**Figure 15:**
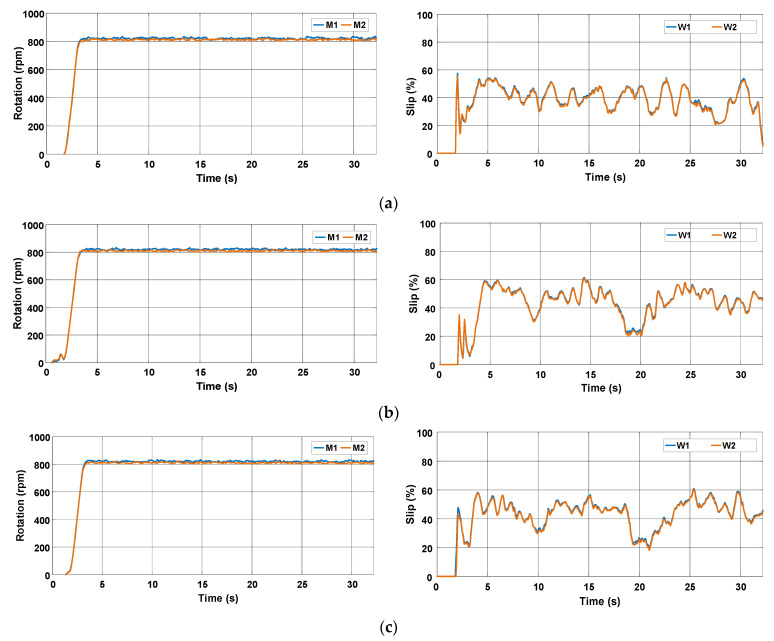
Rotation speed of electric motors and wheel slip during the trial sessions carried out on firm soil and without slip control. (**a**) T#1.1; (**b**) T#1.2; (**c**) T#1.3.

**Figure 16:**
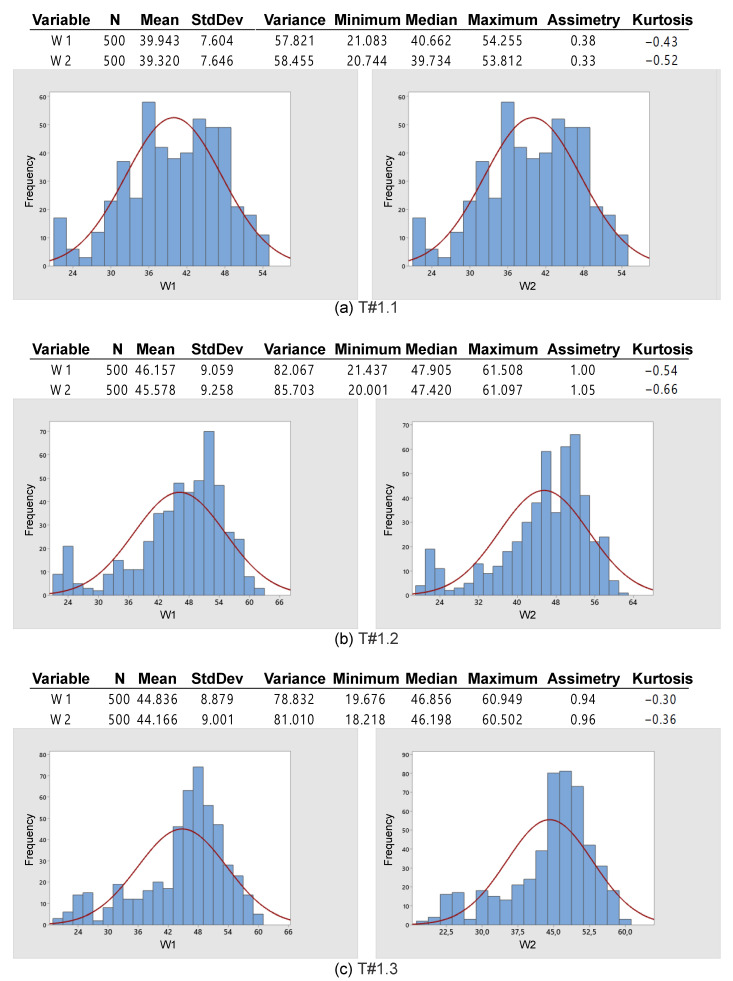
Basic descriptive statistics applied to data obtained during the trial sessions carried out on firm soil and without slip control.

Figure 15 shows the behavior of the rotation speed and slip during the three tests. It is observed that the rotation speed of motors M1 and M2 remains nearly constant as desired, but the slip of the rear wheels is high. Figure 16 represents the data in terms of basic descriptive statistics and histograms (normal curve) related to the slip of the rear wheels W1 and W2. The mean slip ranges from 39.32% to 46.16% without active control. In turn, the standard deviation is between 7.6% and 9.26%. Thus, it is evident that the slip is much higher than the limits recommended for activities on firm ground (8% to 10%). Figure 16 also denotes that the symmetry is between −1.05 and −0.33, whereas the kurtosis is between −0.52 and 0.66. It is then reasonable to state that the data are normally distributed according to [31].

**Figure 17:**
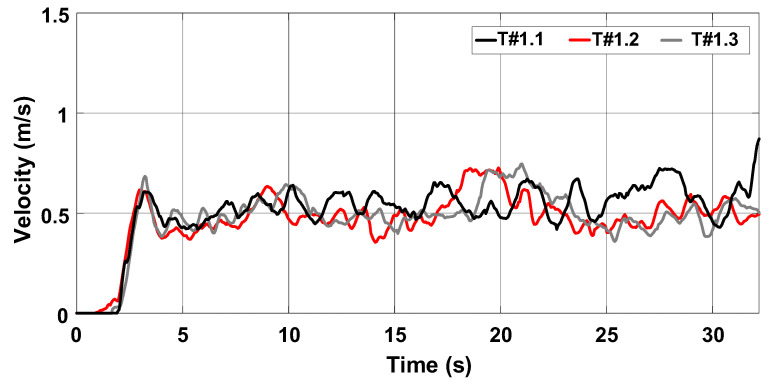
Velocity of the electric tractor during the trial sessions carried out on firm soil and without slip control.

**Figure 18:**
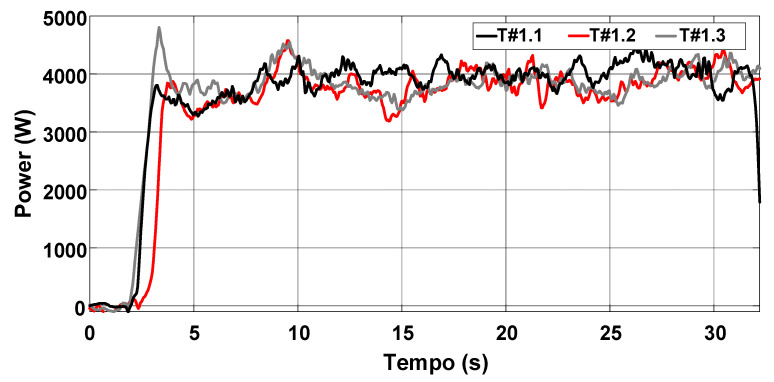
Power consumption during the trial sessions carried out on firm soil and without slip control.

**Figure 19:**
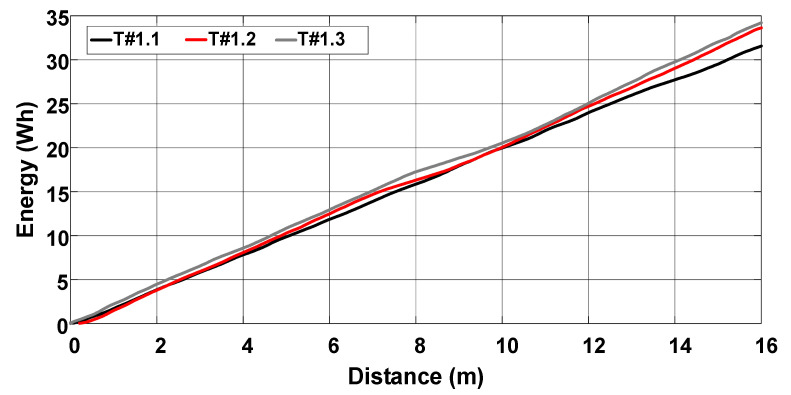
Energy consumption during the trial sessions carried out on firm soil and without slip control.

Figure 17 shows that the average velocity of the electric tractor varies between 0.49 m/s and 0.55 m/s. The current drawn from the battery bank is between 79.28 A and 83.43 A, corresponding to an active power between 3790.68 W and 3968.68 W according to Figure 18. The energy consumption varies between 29.5 Wh and 32 Wh during the 15 m course according to the profile represented in Figure 19. The main parameters measured during the trial sessions are summarized in Table 1.

### 3.2. Test on Firm Soil with Slip Controlled by the Operator

**Figure 20:**
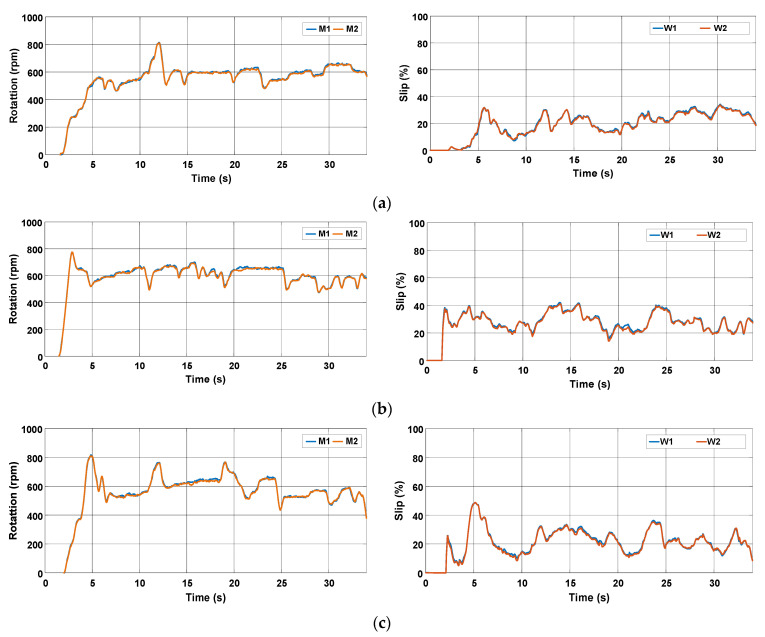
Rotation speed of electric motors and wheel slip during the trial sessions carried out on firm soil with the slip manually controlled by the operator. (**a**) T#2.1; (**b**) T#2.2; (**c**) T#2.3.

In this test, the maximum rotation speed of the motors was set to 800 rpm, but the resultant velocity depends on the manual adjustment made by the operator using the accelerator pedal. Figure 20 shows that the rotation speed of the rear wheels varies according to the operator’s action in an attempt to mitigate the slippage during the tests. Despite the slip decreasing slightly when compared with the previous case, it is still above the range recommended for firm soil operation, that is, between 8% and 10%.

**Figure 21:**
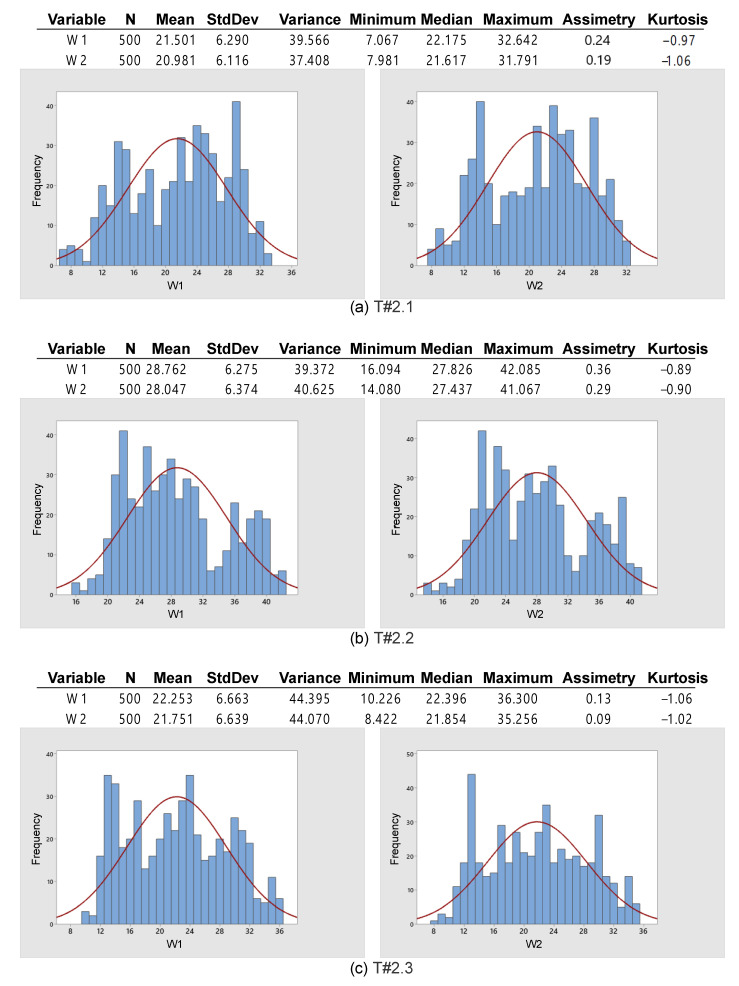
Basic descriptive statistics applied to data obtained during the trial sessions carried out on firm soil with the slip manually controlled by the operator.

Figure 21 presents some figures of merit that allow for evaluating the acquired data statistically. The mean varies between 20.98% and 28.76%, whereas the standard deviation ranges between 6.11% and 6.66%. Thus, it becomes evident that the slip is still much higher than the recommended values. The symmetry is between −0.24 and 0.36, whereas the kurtosis is between −1.06 and −0.89. Once again, the data are arranged in the form of a normal distribution [31].

**Figure 22:**
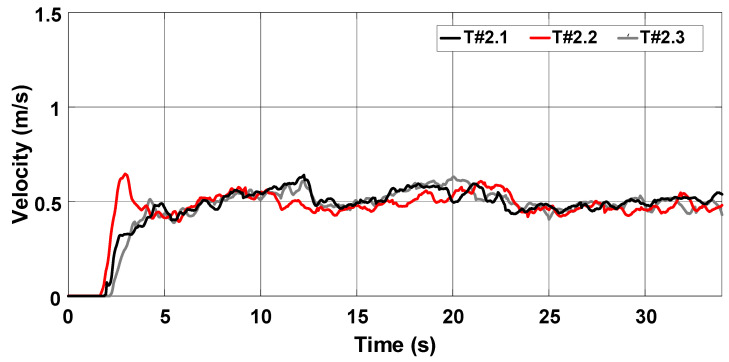
Velocity of the electric tractor during the trial sessions carried out on firm soil with the slip manually controlled by the operator.

**Figure 23:**
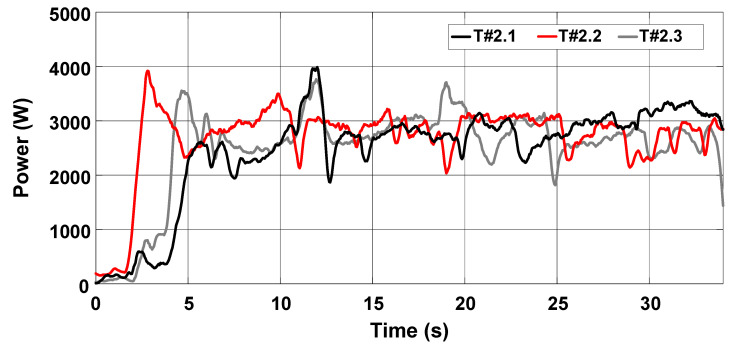
Power consumption during the trial sessions carried out on firm soil with the slip manually controlled by the operator.

**Figure 24:**
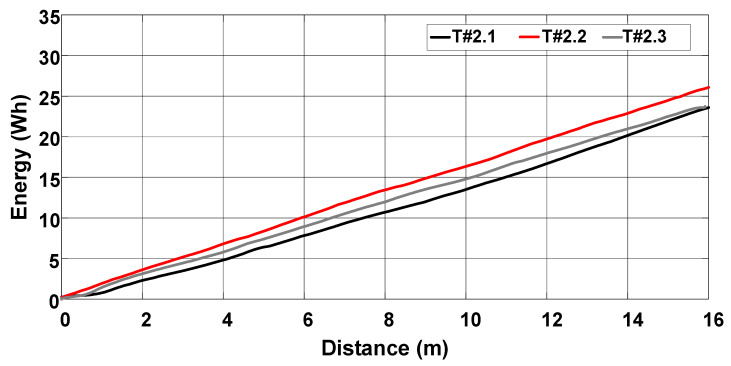
Energy consumption during the trial sessions carried out on firm soil with the slip manually controlled by the operator.

The average velocity of the electric tractor during tests T#2.1, T#2.2, and T#2.3 varied between 0.49 m/s and 0.52 m/s, as shown in Figure 22. The current drawn from the battery ranges between 57.05 A and 59.54 A in Figure 23, resulting in an average power varying between 2742.94 W and 2854.64 W. The resulting energy consumption in Figure 24 varies between 21.94 Wh and 24.51 Wh. Table 2 summarizes the main parameters associated with the data recorded during the tests.

### 3.3. Test on Firm Soil with Slip Control

**Figure 25:**
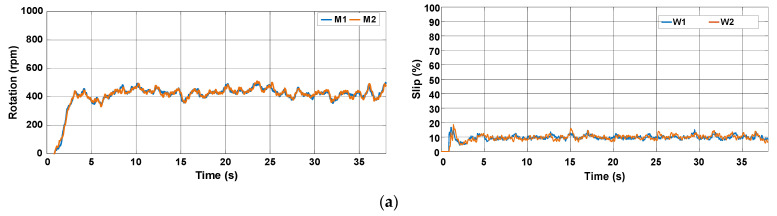

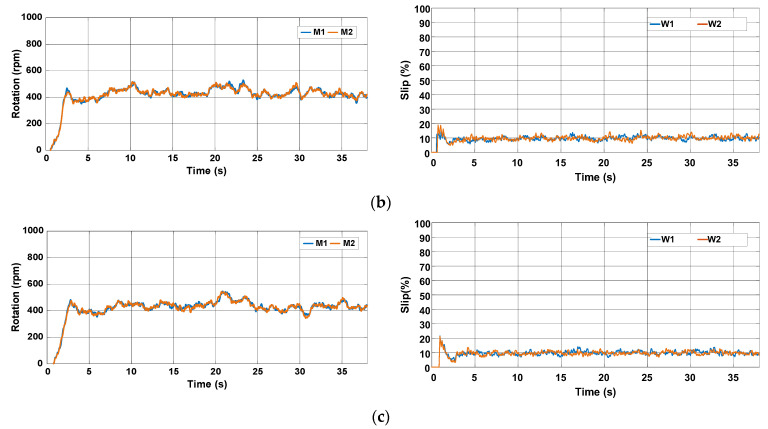
Rotation speed of electric motors and wheel slip during the trial sessions carried out on firm soil with the slip automatically adjusted by the proposed control scheme. (**a**) T#3.1; (**b**) T#3.2; (**c**) T#3.3.

In this test, the slip is adjusted automatically by the proposed control approach. It is worth mentioning that no other solutions typical of ICE-based tractors like ballasting were adopted to improve the tractive efficiency. Figure 25 presents the behavior of the rotation speed and slip during the tests. It is observed that the rotation speed varies, but the slip remains nearly constant.

**Figure 26:**
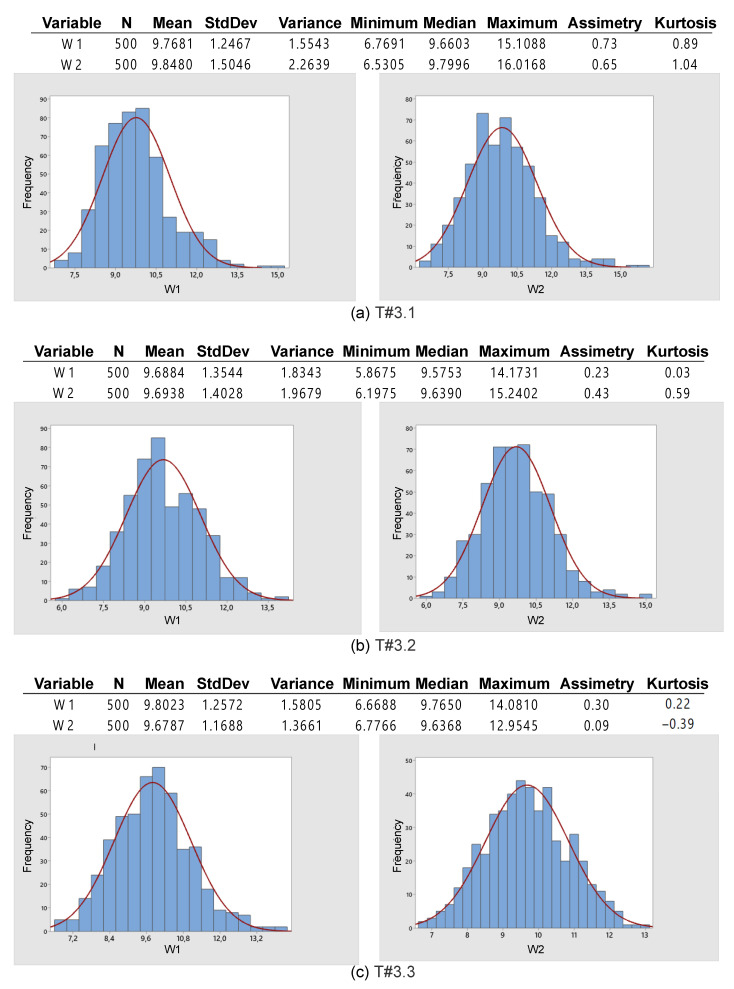
Basic descriptive statistics applied to data obtained during the trial sessions carried out on firm soil with the slip automatically adjusted by the proposed control scheme.

Figure 26 shows that the mean slip remains between 9.68% and 9.85%, which is very close to the desired value. The standard deviation varies between 1.17% and 1.5%, with these values being much lower than those obtained in the previous tests.

Besides the basic descriptive statistics, SCP was also used to perform a more in-depth analysis based on control charts. According to [33], a control chart represents a way of examining variability in time-oriented data. The graph contains a centerline that corresponds to the average value of the controlled and monitored variable. It also contains two other horizontal lines, called the upper control limit (UCL) and lower control limit (LCL). They consist of a pair of statistically derived thresholds that reflect inherent or natural variability in the process, considering three times the standard deviation of concentration values are above and below the centerline. Thus, control charts statistically define UCL and LCL according to Equations (24) and (25), respectively.

(24) LCL=μ−3σ,

(25) UCL=μ+3σ,

where *μ* is the mean and *σ* is the standard deviation.

**Figure 27:**
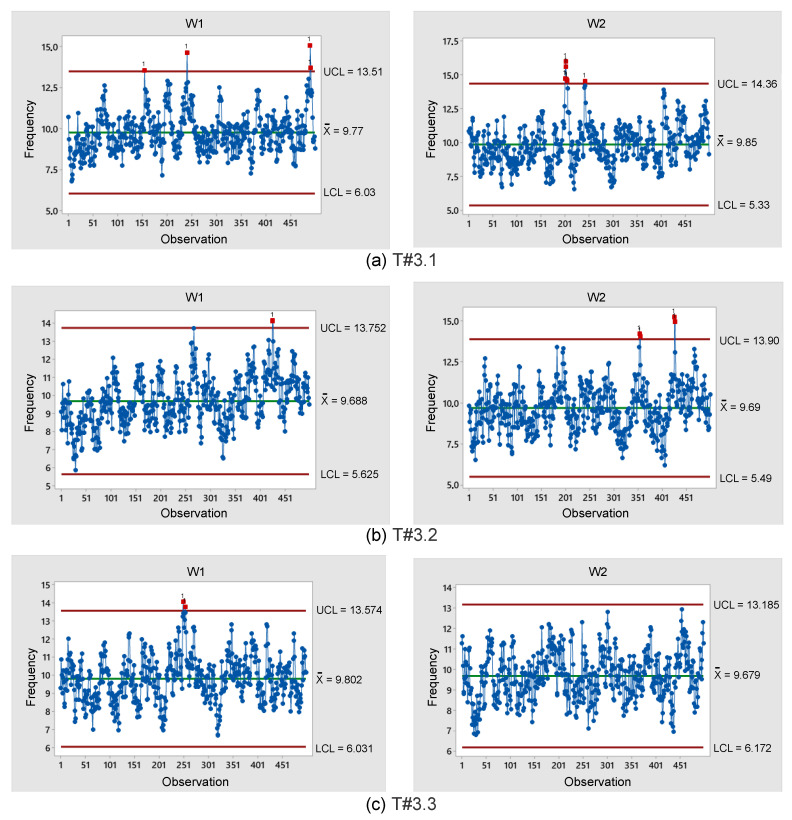
Control charts representing data obtained during the trial sessions carried out on firm soil with the slip automatically adjusted by the proposed control scheme.

If the process operates accordingly without any variability source, the concentrations should vary randomly around the centerline while remaining within the desired control limits [33]. Figure 27 shows the control charts associated with the electric tractor using the proposed slip control.

According to Figure 26, since the symmetry is between 0.09 and 0.73, while the kurtosis is between −0.39 and 1.04, the data are represented by a normal distribution [31]. Figure 27 also evidences that at most five points exceed UCL, representing only 1% of the overall data corresponding to 500 points. According to [33], if only 5% of data are outside the control limits, the process can be considered stable. This assumption is of major importance, especially because the field tests are subject to the most varying conditions in terms of weather, soil, mechanized systems, quality indices associated with agricultural activities, among other aspects. Therefore, it is reasonable to state that the proposed slip control has presented prominent results.

**Figure 28:**
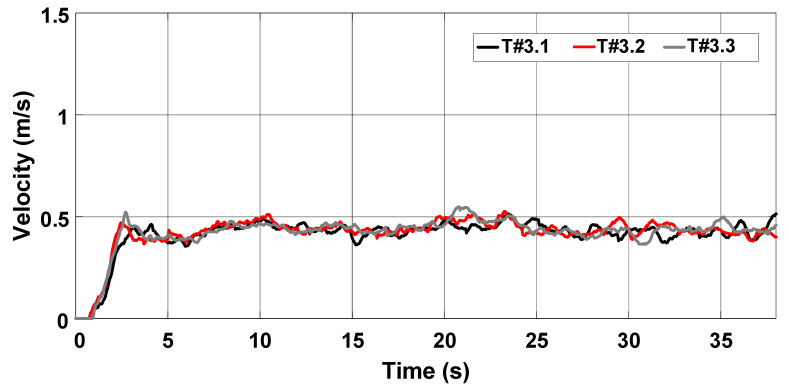
Velocity of the electric tractor during the trial sessions carried out on firm soil with the slip automatically adjusted by the proposed control scheme.

**Figure 29:**
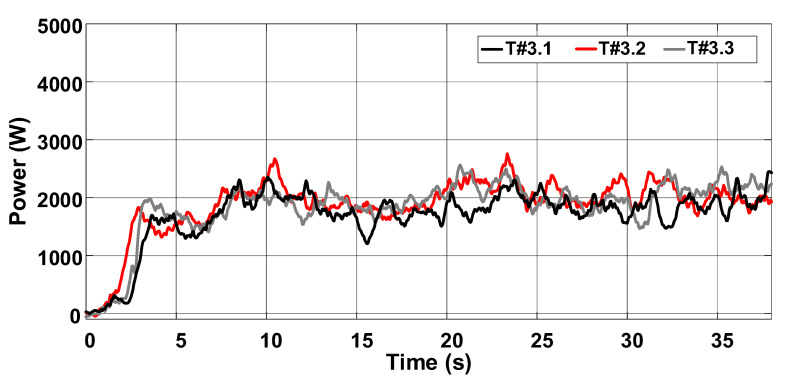
Power consumption during the trial sessions carried out on firm soil with the slip automatically adjusted by the proposed control scheme.

**Figure 30:**
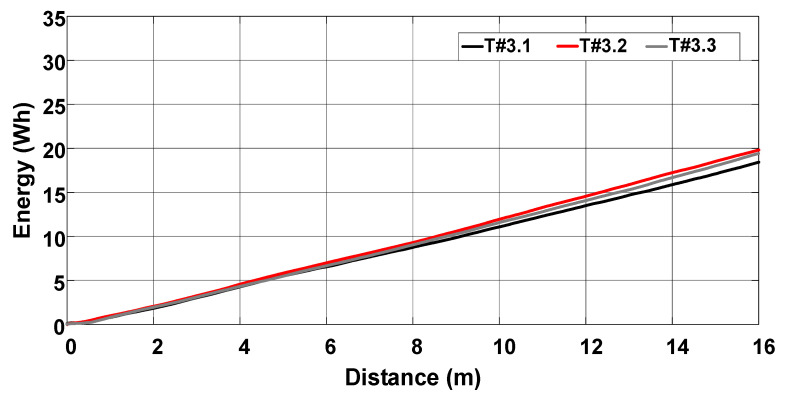
Energy consumption during the trial sessions carried out on firm soil with the slip automatically adjusted by the proposed control scheme.

Figure 28 represents the average velocity, which remained around 0.44 m/s during tests T#3.1, T#3.2, and T#3.3. The average current drawn from the battery bank ranges between 37.27 A and 40.09 A, corresponding to an average power varying between 1820.18 W and 1955.25 W in Figure 29. Figure 30 shows the energy consumption as a function of the traveled distance, which remained nearly constant during the tests. Table 3 also summarizes the main parameters measured with the slip control enabled.

## 4. Conclusions

This work has presented an automatic slip control approach for electric tractors. The experimental results show that the tractive efficiency increases when the slip control is adopted, also resulting in improved performance and increased autonomy. When the slip control is disabled, the electric tractor behaves as a conventional ICE-based vehicle, thus evidencing that slippage is of major concern for practical applications. Overall, the proposed approach can be regarded as a simple solution in terms of the system identification procedure and controller used in the application. It also provides the tractor with a more stable operation, with minimum intervention of the operator during agricultural activities.

It is worth mentioning that the electric tractor presented an average velocity between 0.49 m/s and 0.55 m/s, as well as a mean slip ranging between 39.32% and 46.16%. In turn, the slip control system allows the tractor to achieve an average velocity of 0.44 m/while keeping the slip within the desired range traveling on firm soil, which is between 9.68% and 9.85%. Considering that each type of soil has a proper slip range for which the tractive efficiency is maximum, one can properly adjust the slip so that the control system can always ensure the operation under optimal conditions.

It is reasonable to state that the average velocity remains nearly constant when the slip control is enabled, resulting in lower energy consumption between 17.1 Wh and 18.55 Wh, corresponding to energy savings of around 42%. The proposed technique also allows for mitigating the operator fatigue, while also contributing to the increase of tractive efficiency during agricultural activities. Overall, the results corroborate the hypothesis that distributed electric propulsion systems using two three-phase induction motors and an ECU specifically designed for small electric tractors are capable of providing good technical performance and operational flexibility.

## Figures and Tables

**Table 1:** Summary of parameters measured during the trial sessions carried out on firm soil and without slip control.

Parameter	T#1.1	T#1.2	T#1.3
Execution time (15 m) (s)	27.27	30.61	29.41
Velocity (m/s)	0.55	0.49	0.51
Velocity (km/h)	1.98	1.76	1.94
Mean slip (W1/W2) (%)	39.92/39.94	46.16/45.58	44.84/44.17
Average current drawn from batteries (A)	83.43	79.28	81.36
Average power drawn from batteries (W)	3968.68	3790.68	3889.88
Energy consumption (Wh)	29.5	31.34	32

**Table 2:** Summary of parameters measured during the trial sessions carried out on firm soil firm soil with the slip manually controlled by the operator.

Parameter	T#2.1	T#2.2	T#2.3
Execution time (15 m) (s)	29.41	30.61	28.85
Velocity (m/s)	0.51	0.49	0.52
Velocity (km/h)	1.83	1.76	1.87
Mean slip (W1/W2) (%)	21.5/21.98	28.76/28.05	22.25/21.75
Average current drawn from batteries (A)	57.05	59.54	57.50
Average power drawn from batteries (W)	2742.94	2854.64	2766.99
Energy consumption (Wh)	21.94	24.51	22.5

**Table 3:** Summary of parameters measured during the trial sessions carried out on firm soil firm soil with the slip automatically adjusted by the proposed control scheme.

Parameter	T#3.1	T#3.2	T#3.3
Execution time (15 m) (s)	34.1	34.1	34.1
Velocity (m/s)	0.44	0.44	0.44
Velocity (km/h)	1.58	1.58	1.58
Mean slip (W1/W2) (%)	9.77/9.85	9.69/9.70	9.80/9.68
Average current drawn from batteries (A)	37.27	40.09	39.63
Average power drawn from batteries (W)	1820.18	1955.25	1931.35
Energy consumption (Wh)	17.1	18.55	18

## Data Availability

Data available on request from the authors.
